# Antibacterial Activity of Ulva/Nanocellulose and Ulva/Ag/Cellulose Nanocomposites and Both Blended with Fluoride against Bacteria Causing Dental Decay

**DOI:** 10.3390/polym15041047

**Published:** 2023-02-20

**Authors:** Ragaa A. Hamouda, Fauzia A. K. Qarabai, Fathi S. Shahabuddin, Turki M. Al-Shaikh, Rabab R. Makharita

**Affiliations:** 1 Department of Biology, College of Sciences and Arts at Khulis, University of Jeddah, Jeddah 21959, Saudi Arabia; 2 Department of Microbial Biotechnology, Genetic Engineering and Biotechnology Research Institute (GEBRI), University of Sadat City, Sadat City 32897, Egypt; 3 Harad Center Makkah Ministry of Health, Riyadh 24342, Saudi Arabia; 4 Botany and Microbiology Department, Faculty of Science, Suez Canal University, Ismailia 41522, Egypt

**Keywords:** *Ulva lactuca*, *S. mutans* ATCC 25175, *L. acidophilus* CH-2, cellulose, nanocellulose

## Abstract

One of the most prevalent chronic infectious disorders is tooth decay. Acids produced when plaque bacteria break down sugar in the mouth cause tooth decay. *Streptococcus mutans* and *Lactobacillus acidophilus* are the most prominent species related to dental caries. Innovative biocidal agents that integrate with a biomaterial to prevent bacterial colonization have shown remarkable promise as a result of the rapid advancement of nanoscience and nanotechnology. In this study, *Ulva lactuca* was used as a cellulose source and reducing agent to synthesize nanocellulose and Ulva/Ag/cellulose/nanocomposites. The characterizations of nanocellulose and Ulva/Ag/cellulose/nanocomposites were tested for FT-IR, TEM, SEM, EDS, XRD, and zeta potential. *Ulva*/Ag/cellulose/nanocomposites and Ulva/nanocellulose, both blended with fluoride, were tested as an antibacterial against S. mutans *ATCC 25175* and *L. acidophilus CH-2.* The results of the SEM proved that nanocellulose is filament-shaped, and FT-IR proved that the functional groups of Ulva/nanocellulose and *Ulva*/Ag/cellulose/nanocomposites and cellulose are relatively similar but present some small diffusion in peaks. The TEM image demonstrated that the more piratical size distribution of *Ulva*/Ag/cellulose/nanocomposites ranged from 15 to 20 nm, and *Ulva*/nanocellulose ranged from 10 to 15 nm. Ulva/Ag/cellulose/nanocomposites have higher negativity than *Ulva*/nanocellulose. *Ulva*/Ag/cellulose/nanocomposites and *Ulva*/nanocellulose possess antibacterial activity against *S. mutans ATCC 25175* and *L. acidophilus CH-2*, but Ulva/Ag/cellulose/nanocomposites are more effective, followed by that blended with fluoride. It is possible to use Ulva/Ag/cellulose/nanocomposites as an antimicrobial agent when added to toothpaste. It is promising to discover an economic and safe nanocomposite product from a natural source with an antimicrobial agent that might be used against tooth bacteria.

## 1. Introduction

One of the most significant problems in public health is tooth decay [1]. General health is affected by oral health [2]. Dental caries is related to behavioral, economic, and social aspects and increases the incidence of diseases among people [3]. Dental caries is a bacterial illness that gradually arises from complicated biological interactions between acidogenic bacteria and fermentable carbohydrates [4]. Acids from bacterial metabolism that diffuse into enamel and dentine generate the bacterial disease process known as dental caries, which is contagious [5]. Worldwide, 36% of people suffer dental caries in their adult teeth (Gram-positive bacteria creating tooth decay) [6]. Dental caries is caused by *Streptococcus mutans*, and this requires that the organism can build biofilms and create acid on the tooth surface [7]. These bacteria are extremely acidogenic, creating short-chain acids that tenderize the hard tissues of teeth [8]. They adhere tightly to the surface of the teeth by synthesizing insoluble carbohydrates with three isozymes of glucosyl transferases, catalyzing and metabolizing sucrose [9]. *Streptococcus mutans* and Lactobacilli are most connected with dental caries [10]. Dental caries and necrotizing fasciitis are just two of the illnesses that streptococci can cause (ibid.). They are the sole bodily portion that is not subject to metabolic change; tooth surfaces are special [11].

Nanotechnology is significant in biomedical applications as a different antimicrobial strategy due to the recurrence of diseases and antibiotic-resistant bacteria [12]. The development of a new area dubbed “nano dentistry” is being fueled by the rising interest in dental uses of nanotechnology [13]. Furthermore, nanoparticles have antimicrobial coatings and films as alternative methods to prohibit biofouling [14].

A linear polysaccharide polymer known as cellulose is found in various biomasses, including cotton, trees, tunicates, algae, plants, and trees [15]. Green algae are the best natural source for extracting cellulose [16]. Cellulose can be processed chemically, mechanically, or enzymatically to create nanocellulose [17]. Nanocellulose is made up of 1:100 nm-sized cellulose fibrils [18].

Cellulose nanofiber (NFC), also referred to as nanocellulose, is a rapidly developing source of “green technology”, “recyclable”, “renewable”, “eco-friendly”, “triggered biodegradable”, and “sustainable” materials [19]. Nanocellulose is preferable to cellulose due to its high aspect ratio and large surface area [20]. Nanocellulose is divided into three classes: nanocrystalline celluloses (CNC), nanofibrillated celluloses (NFC), and bacterial celluloses (BC) [21]. These three divisions are based on sources, functions, modes of production, structures, and reaction conditions [22]. Nanocellulose has become widely used in many applications due to its good mechanical properties, renewable susceptibility, and role in improving composites [23]. Due to its biocompatibility, biodegradability, and nontoxicity, nanocellulose is used as an antimicrobial in biomedical applications [24].

Cellulose nanofibers are used to produce nanocomposites from inorganic compounds due to their high specific surface area and small size [25]. ZnO/CNC presented enhanced antibacterial activity when compared with pure ZnO against both *S. aureus* and *E. coli* [26]. Moreover, ZnO/BC composites have demonstrated vigorous antibacterial activity against *S. aureus* and *E. coli* [27]. Synthesized Ag@AgCl-reinforced cellulose composites demonstrated enhanced antibacterial activities [28]. Fe_3_O_4_/Ag@NFC nanocomposites can also be employed in medicinal applications as an antibacterial substance [29]. Nanocomposites consist of cellulose, AgCl, and Ag successfully synthesized with excellent antimicrobial properties [30].

Fluoride has been used to prevent cavities for about forty years, and during the past twenty years, it has been used more widely [31]. Fluoride works by three mechanisms to prevent decay: inhibiting bacterial enzymes, reinforcing remineralization at crystal surfaces, and preventing purification on crystal surfaces [32]. Proper use of fluoride improves oral health and promotes general health [33]. In the late 1970s, the first signs of fluoride’s positive effect on children’s dental health appeared in the UK [34]. The daily utilization of fluoride toothpaste is a major reason for the decline in the rate of caries around the world [35]. Persistent low-level treatments of fluoride are more effective in caries protection than rare usage of high-level treatments [36]. The status of water fluoridation in caries prohibition is obvious around the world [37].

This research aims to extract cellulose from marine alga (*Ulva lactuca),* synthesize cellulose nanoparticles, and perform biosynthesis of Ulva/Ag/cellulose nanocomposites. Furthermore, it aims to investigate the antibacterial activities of cellulose nanoparticles and Ulva/Ag/cellulose nanocomposites and both blended with fluoride to provide reinforcement against *Streptococcus mutans* and *Lactobacillus acidophilus*, which cause tooth decay.

## 2. Materials and Methods

### 2.1. Materials

The materials used in the study were green alga (*Ulva lactuca*), sodium hydroxide (NaOH), ethanol (99%), hydrochloric acid (37%), hydrogen peroxide (6%), silver nitrate 99.9+% (metals basis), and distilled water. All the chemicals used in this research were of analytical grade and applied without further purification. Chemical materials were purchased from the Saudi Chemical company (PanReac AppliChem, Ar Riyad, Saudi Arabia).

### 2.2. Alga Collection

Alga was collected from the Red Sea shore in Jeddah, Saudi Arabia (21°38′43.4″ N 39°06′04.7″ E). To remove impurities, alga was rinsed in water and then dried in an oven set to 60 °C. Crushed and sieved dry samples were used.

### 2.3. Extracting Cellulose from U. lactuca Green Alga

*Ulva lactuca* (50 g) was milled to a soft powder. After that, 50 g of grounded alga was placed in a flask with 170 mL of pure ethanol and 30 mL of water for 6 h over heat at 60 °C with a magnet (stirring) and filtered, and the liquid phase was discarded. The insoluble fraction was repeatedly cleaned with 99% ethanol before being dried for 16 h at 37 °C in the oven. After drying, the sample was further processed by suspending it in 400 mL of 4% H_2_O_2_, which was then heated to 80 °C for 16 h to remove any remaining green pigments and other colored impurities. The mixture was filtered, and the liquid phase was discarded after cooling to room temperature. The insoluble fraction was then suspended in 400 mL of 0.5 M Na OH after being rinsed with distilled water. For 16 h, the mixture was maintained at 60 °C and was then taken out of the oven, allowed to cool to room temperature, filtered, and washed three times with distilled water before the insoluble fraction was collected.

### 2.4. Extraction of Nanocellulose

The extracted cellulose was treated with 27 mL HCl and 173 mL of distilled water at 90 °C for 10 min. The combination was filtered by removing the liquid phase after cooling to room temperature, and the insoluble fraction was then cleaned with distilled water. When the mixture was entirely dry, it was stored at 60 °C.

### 2.5. Synthesis Ulva/Ag/Cellulose Nanocomposite

#### 2.5.1. Preparation of Silver Nanoparticles

##### Algal Extract

Dry alga *Ulva lactuca* (1 g) was added to 100 mL distilled water, boiled for 1 h, and then filtered.

##### Biosynthesis of Ag Nanoparticles

Silver nitrate (0.17 g) was added to 90 mL of distilled water. The prepared alga extract (10 mL) was added drop-wise to the solution at 60 °C with constant stirring until the mixture turned brown.

#### 2.5.2. Biosynthesis of Ulva/Ag/Cellulose Nanocomposites

Silver nitrate (0.085 g) was added to 45 mL of distilled water, then different concentrations of nanocellulose (0.1, 0.2, 0.4, and 0.8 g) were added separately, and then alga extract (5 mL) was added as drops. The solution was heated at 60 °C and stirred until the color changed to brown.

#### 2.5.3. Biosynthesis of Ulva/Nanocellulose, Ulva/Ag/Cellulose Nanocomposites, and Both Blended with Fluoride

Silver nanoparticles (1.7 mg/mL) were synthesized by *U. lactuca*, 2 mg/mL Ulva/nanocellulose, and 2 mg/mL Ulva/Ag/cellulose nanocomposites, and 1 mL of each was blended with 10 mL fluoride (1.23%) by magnetic stirrer for 10 min.

### 2.6. Characterization

#### 2.6.1. Fourier Transform Infrared (FT-IR)

Ulva/cellulose and Ulva/Ag/cellulose nanocomposite functional groups were examined using a Fourier transform infrared spectrometer (FT-IR), Thermo Fisher Nicolet IS10, (Waltham, MA, USA) Spectrometer, FT-IR spectrum ranged between 4000 and 400 cm^−1^.

#### 2.6.2. Scanning Electron Microscope (SEM)

The morphologies of Ulva/nanocellulose and Ulva/Ag/cellulose nanocomposite were examined using scanning electron microscopy (SEM) operating at 30 kV (SEM, JEOL JSM-6510/v, Tokyo, Japan).

#### 2.6.3. Transmission Electron Microscopy (TEM)

The morphology of synthesized Ulva/nanocellulose and Ulva/Ag/cellulose nanocomposite were examined using TEM (JEOL JSM-6510/v, Tokyo, Japan) at the nanoscale.

#### 2.6.4. X-ray Powder Diffraction (XRD)

X-ray diffraction patterns of Ulva/nanocellulose and Ulva/Ag/cellulose nanocomposite were analyzed using an X-ray diffractometer (PAN Analytical X-Pert PRO, spectris plc, Almelo, The Netherlands). The cellulose size was determined using Scherrer’s equation.

*Crystal Size L = λk/c β θ*

where *λ* = 0.1540 nm, *k* is the constant factor of 0.91, *θ* = diffraction angle in radians, and *β* = full width at half maximum (FWHM).

#### 2.6.5. Energy-Dispersive Spectroscopy (EDS)

A field emission scanning electron microscope equipped with energy-dispersive spectroscopy (EDS) (JEOL JSM-6510/v, Tokyo, Japan) was used to investigate the surface morphology of the Ulva/nanocellulose and Ulva/Ag/cellulose nanocomposite.

#### 2.6.6. Zeta Potential Analysis

The zeta potential of the Ulva/nanocellulose and Ulva/Ag/cellulose nanocomposite provides details of the stabilization in the middle of the liquid that it is dispersed in (Malvern Zeta size Nano-Zs90, Malvern, PA, USA).

#### 2.6.7. Differential Scanning Calorimetry (DSC)

Differential scanning calorimetry DSC testing 19 mg of Ulva/Ag/cellulose nanocomposite blend with fluoride was conducted using differential scanning calorimeter DSC131 EVO France.

### 2.7. Antibacterial Activities

The agar-well diffusion technique was used to study the antibacterial properties of hybrids made of AgNPs, Ulva/nanocellulose, and Ulva/Ag/cellulose nanocomposite, and both blended with fluoride against *S. mutans* ATCC 25175 and *L. acidophilus* CH-2 [38] as model Gram-positive bacteria associated with dental caries. Muller–Hinton agar was poured into a Petri dish and solidified. The turbidity of an overnight broth culture of *S. mutans* ATCC 25175 and *L. acidophilus* CH-2 was adjusted to 0.5 McFarland standards. Then, 50 µL of bacterial suspension was spread over the plates separately using a sterilized cotton swab. After that, 100 µL of the prepared nanoparticles at different concentrations of nanocellulose (0.2, 0.4, 0.8, and 1.6%) was applied to 0.7 mm-diameter wells on each bacterial agar plate. Plates were incubated at 37 °C for 24 h. The inhibitory zone (mm) was recorded. The experiment was conducted in triplicate.

### 2.8. Minimum Inhibitory Concentration (MIC)

The minimum inhibitory concentration (MIC) of Ulva/Ag/cellulose nanocomposite was examined using the standard broth dilution method at diverse concentrations oscillating from 0.002 to 0.00005 g/mL. Mueller–Hinton agar (MHA) medium was prepared and inoculated under aseptic conditions with 50 μL of the overnight bacteria suspension and allowed to dry. Wells were filled with 100 μL of different serial dilutions of Ulva/Ag/cellulose nanocomposite separately. After 24 h of incubation at 37 °C, plates were checked to see if an inhibition zone (mm) had formed around each well.

### 2.9. Statistical Analysis

Data were obtainable as mean ± SEM, and SPSS 16 was used for the statistics, together with one-way ANOVA.

## 3. Results and Discussion

### 3.1. FT-IR Spectroscopy Analysis

**Figure 1:**
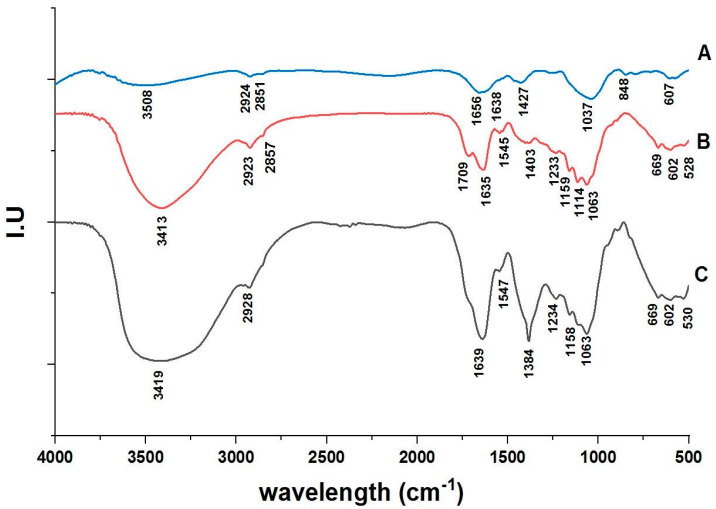
FT-IR spectroscopy of Ulva cellulose (**A**), Ulva nanocellulose (**B**), and Ag/Ulva cellulose nanocomposites (**C**).

Figure 1 and Table 1 show the results of the FT-IR spectroscopy analysis of cellulose, nanocellulose, and Ulva/Ag/cellulose nanocomposite derived from *U. lactuca*. The results demonstrate that 9 peaks were obtained with cellulose, 18 peaks with nanocellulose, and 11 peaks with Ag/Ulva cellulose nanocomposites. The results investigated the modification of numbers and positions (wavenumbers) and demonstrated the differences in the chemical structure of each compound.

### 3.2. SEM and TEM Images

**Figure 2:**
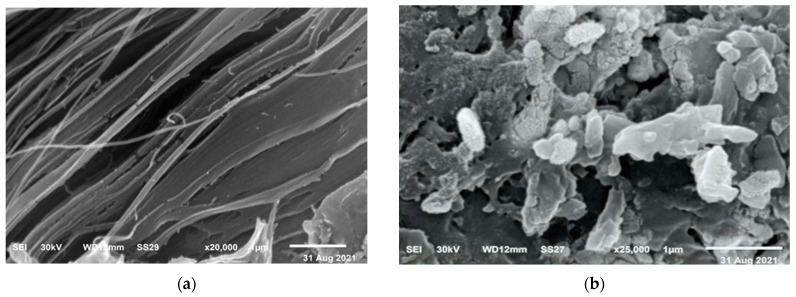
Scanning electron microscopic (SEM) image of biosynthesis: nanocellulose (**a**) and Ag/cellulose nanocomposites (**b**) derived from *U. lactuca*.

**Figure 3:**
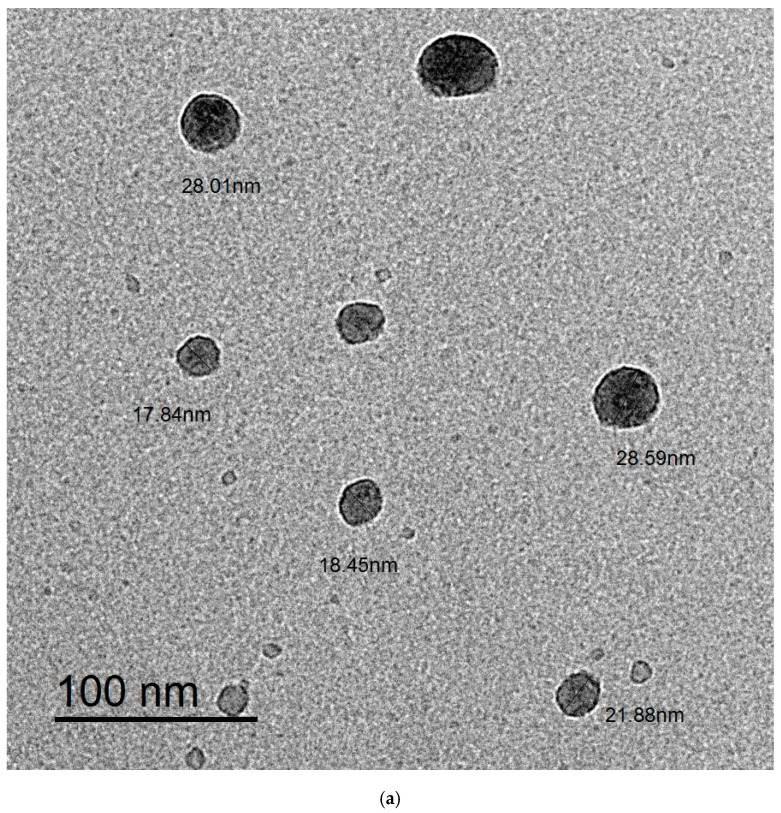

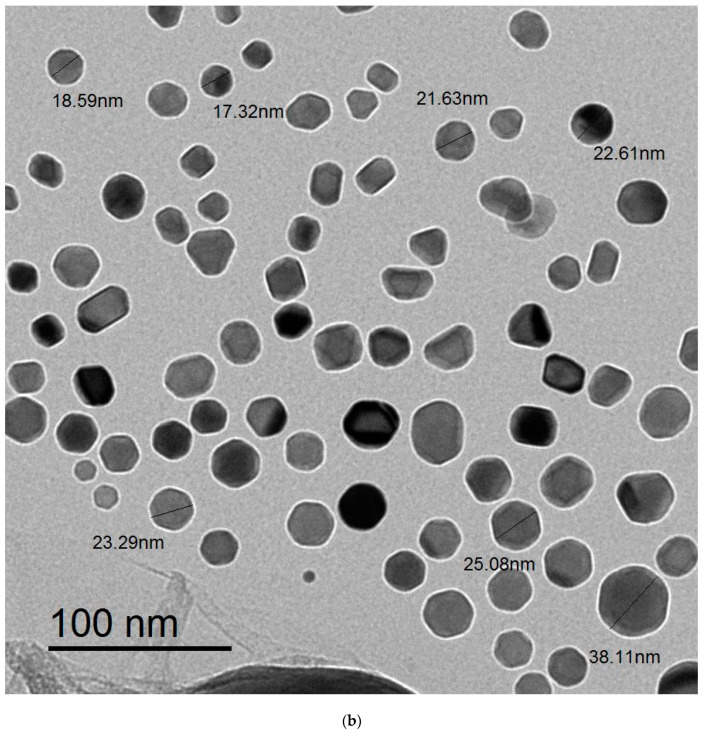
Transmission electron microscopic (TEM) image of biosynthesis: Ulva/nanocellulose (**a**) and Ulva/Ag/cellulose nanocomposites (**b**).

**Figure 4:**
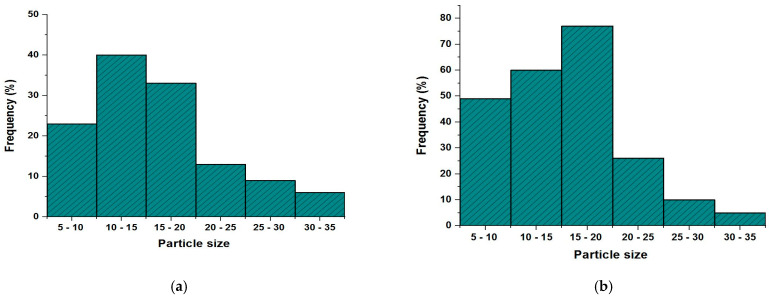
Piratical size distribution of Ulva/nanocellulose (**a**) and Ulva/Ag/cellulose nanocomposites (**b**) derived from *U. lactuca*.

Figure 2a displays SEM (scanning electron microscope) images of the surface of nanocellulose extracted from *U. lactuca*. The image showed that nanocellulose extracted from *U. lactuca* comprises filaments, a result proved by Xiang et al. [63] who reported that the SEM imagery of Cladophora glomerata nanocellulose comprises filaments. Peng et al. [64] demonstrated that nanocellulose is arranged in a fiber network. The SEM image of Ulva/Ag cellulose nanocomposites indicates ridges and valley surfaces (Figure 2b), which confirmed a highly organized by layer porous architecture and large surface area. These results are confirmed by Tan [65], who reported that, after the addition of Ag rough, nonhomogeneous Turing structures increased. Figure 3a–b shows TEM images of biosynthesized nanocellulose and Ulva/Ag/cellulose nanocomposites derived from *U. lactuca*. The morphological studies of Ulva nanocellulose indicate a polydispersive and spherical shape and the major range size is from 10 to 15 nm (Figure 4a). The fine particle size will result in a large surface area that will enhance the nanoparticles’ catalytic activity. The results appeared in TEM images of Ulva/Ag/cellulose nanocomposites that are polydispersed hexagonal-shaped nanoparticles with sizes ranging from 5 to 38.1 nm, however, the major size distribution was in the range of 15–20 nm (Figure 4b). The image clarifies that there is a shell core around the AgNPs, a core nanostructure that appears dark, in which nanocellulose appears as shells around the AgNPs. Capping of metal nanoparticles is one of the critical methods for ensuring its stability, AgNPs synthesized and capped by a secreted polysaccharide–protein matrix of Spirulina platensis were quasispherical shaped nanoparticles captured in a polysaccharide–protein matrix sheath with sizes ranging from 5.04 to 33.56 nm [66]. Rajeshkumar et al.’s [67] TEM results of AgNP-based chitosan nanocomposite found it to be spherical with a size of around 10–60 nm.

### 3.3. Energy-Dispersive X-ray Measurements

**Figure 5:**
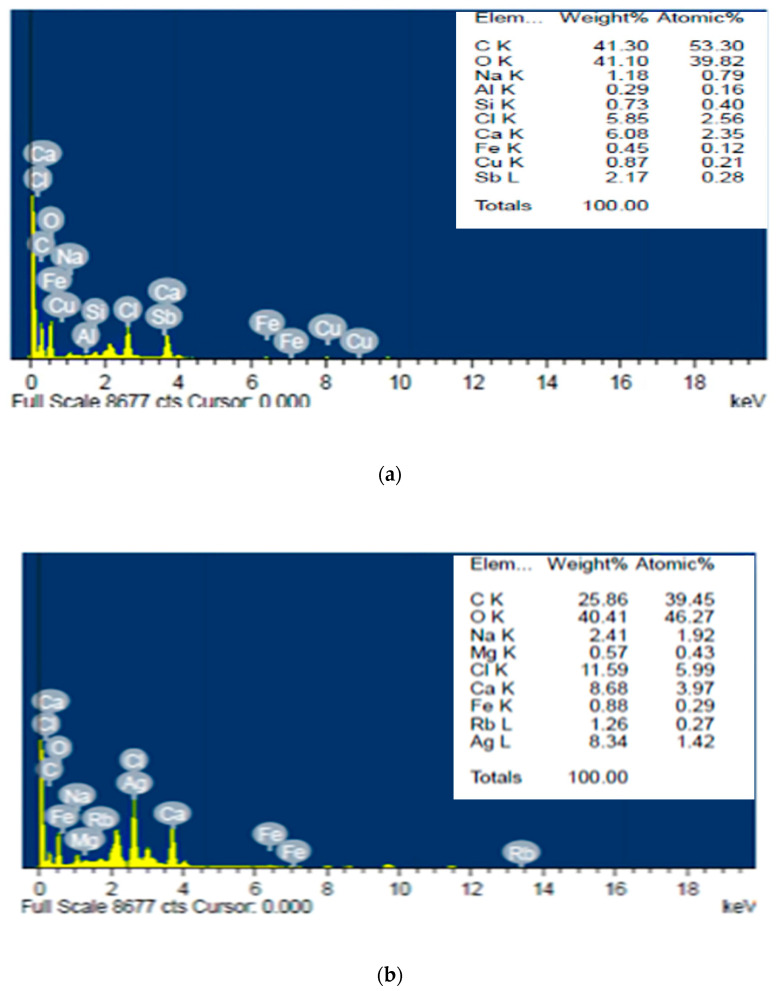
Energy-dispersive X-ray spectrophotometry analysis of biosynthesis: nanocellulose (**a**) and Ulva/Ag/cellulose nanocomposites (**b**) derived from *U. lactuca*.

An analytical method called energy-dispersive X-ray spectroscopy can be used to determine the relative abundance of different elements in a given sample. It depends on a sample and an X-ray excitation source interacting (Figure 5). EDS can be used to identify the chemical elements present in a sample and quantify their relative abundance (qualitative and quantitative analysis). The EDS analysis of nanocellulose derived from *U. lactuca* confirmed that there are nine elements, C, O, Al, Si, Cl, Ca, Fe, Cu, and Sb, with percentage weights of 41.3, 4.1, 0.29, 0.73, 5.85, 6.08, 0.45, 0.87, and 2.17, respectively. Also, there are nine elements presented in Ag/cellulose nanocomposites, C, O, Na, Mg, Cl, Ca, Fe, Rb, and Ag, with percentage weights of 25.86, 40.41, 2.41, 0.57, 11.59, 8.68, 0.88, 1.26, and 8.34%, respectively. The weight of carbon atoms, 41.3% in nanocellulose, became 25.86% in Ag/cellulose nanocomposites, It appears that Ag replaced carbon atoms in Ulva/nanocellulose and formed Ag/cellulose nanocomposites (Figure 4a,b). The analysis of the TiO_2_/CNF composite using energy-dispersive X-ray spectroscopy (EDS) is mostly composed of the three elements, C, O, and Ti [68]. Energy-dispersive X-ray (EDX) analysis was used to emphasize the elemental structure of the silver nanospheres/graphene oxide composite [69]. Silver nanoparticles were well dispersed on the surface of cellulose and penetrated into the cellulose network [70].

### 3.4. X-ray Diffraction

**Figure 6:**
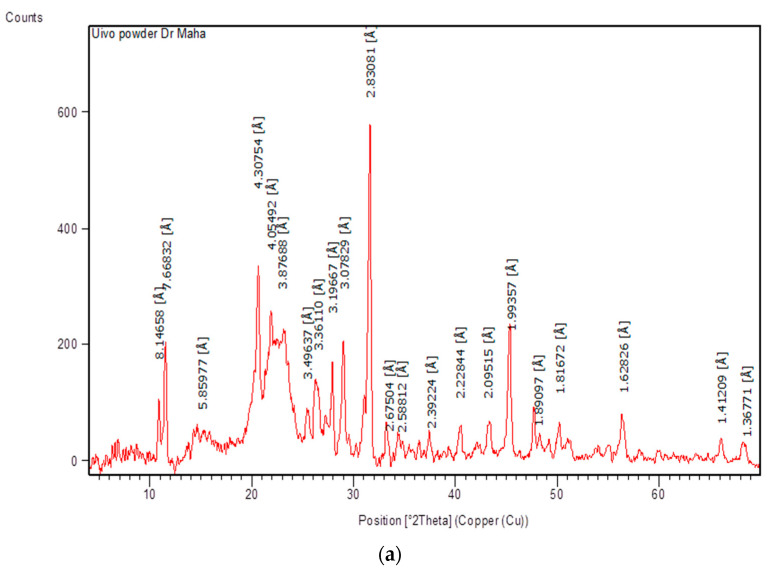

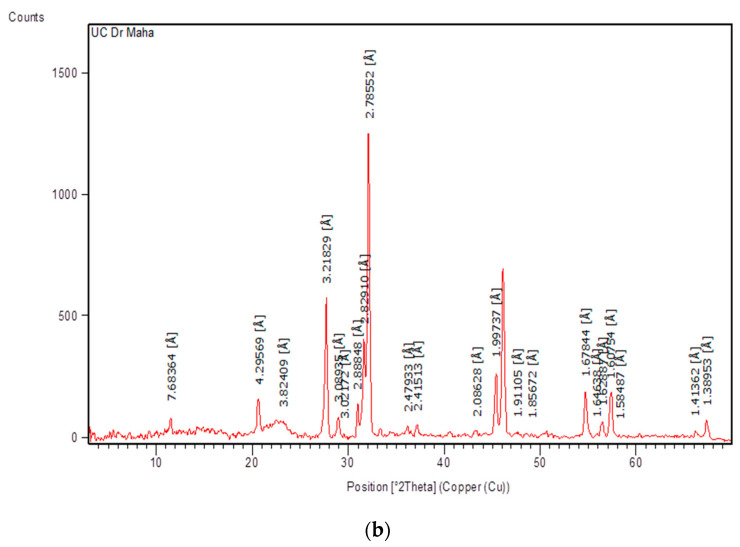
XRD analysis of biosynthesis: nanocellulose (**a**) and Ulva/Ag/cellulose nanocomposites (**b**) derived from *U. lactuca*.

The intensity and shape of the peaks in the XRD patterns are affected by crystal size and crystalline shape. As can be seen, the XRD pattern of nanocellulose and Ulva/Ag/cellulose nanocomposites derived from *U. lactuca* indicate the crystal structure. The XRD diffraction patterns of nanocellulose derived from *U. lactuca* were recorded at 2θ, 10.8, 11.5, 15.1, 20.6, 21.9, 22.9, 25.4, 26.5, 27.9, 29, 31.6, 33.5, 34.6, 37.6, 40.4, 43.1, 45.5, 48.1, and 50.2, which correspond to lattice planes (hkl) (200), (210), (220), (400), (330), (331), (422), (431), (432), (440), (610), (443), (622), (551), (559), (733), (831), (911), and (762) (Figure 6a and Table 2). The major crystalline peak was obtained at 2θ (31.6°) with an intensity of 100% and crystalline size of 27.83, which confirmed that the nanocellulose obtained is nanocrystalline. The peaks at 2θ, 21.9, 22.9, and 45.5 are the broad bands and denote the amorphous nanocellulose. The conventional two-phase cellulose model illustrates cellulose chains as comprising both crystalline (ordered) and amorphous (less ordered) regions [71]. The methods of cellulose synthesis were affected in amorphous and crystalline regions in cellulose nanofibril (CNF) [22]. The XRD peaks of CNCs at 15°, 16.5°, 22.3°, and 34.4° reflect the (100), (110), (200), and (004) planes of cellulose [72]. The peaks of XRD diffraction patterns of Ulva/Ag/cellulose nanocomposites were recorded at 2θ, 20.6, 23.2, 27.7, 28.9, 29.5, 30.9, 31.6, 32.1, 36.2, 37.2, 43.3, 45.4, 47.5, 49.0, 54.6, 55.8, 56.4, 57.3, 58.2, 66.0, and 67.3. The main crystalline peak was obtained at 2θ (32.1°) with an intensity of 100% and crystalline size of 31.35, and all peaks were sharp and all crystalline sizes in nm, which confirmed the crystalline Ulva/Ag/cellulose nanocomposites (Figure 6b and Table 2b). When the diffraction peak is quite sharp, which indicates that the silver has good crystallization performance [73] The diffraction peaks at 38.1°, 44.19°, 64.4°, and 77.4° corresponding to lattice planes (hkl) from the (111), (200), (220), and (311) crystallographic planes of cubic AgNPs [74]. The XRD analysis of neat CNF-Ag NPs composites revealed the presence of Ag nanoparticles with the peaks at 2θ = 37.68°, 43.97°, 64.12° and 77.22° which are corresponding to (111), (200), (220), and (311) in the region from 20 to 80° [75].

### 3.5. Zeta Potential Analysis

**Figure 7:**
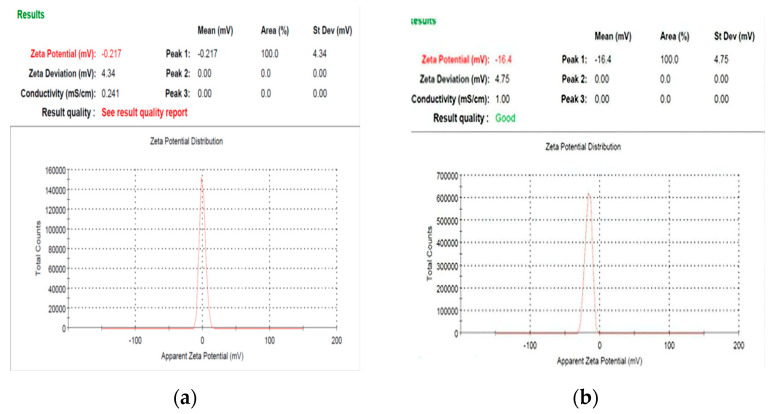
Zeta potential analysis of biosynthesis: nanocellulose (**a**) and Ulva/Ag/cellulose nanocomposites (**b**) derived from *U. lactuca*.

From the zeta potential results presented in Figure 7a,b, it is noticeable that the surface charge of the nanocellulose derived from *U. lactuca* has a negative charge of −0.217 mV, and Ulva/Ag cellulose nanocomposite is−16.4 mV. The results demonstrate that Ulva/Ag cellulose nanocomposites are more negative than Ulva/nanocellulose. Abo-Elmagd et al. [76] stated that charges and strong resistive forces between the particles prevent aggregation and keep the nanoparticles in the medium stable. The zeta potential value of AgNPs photosynthesized by *Oscillatoria limnetica* was −27.4 [77]. The negative charge of nanoparticles revealed the repulsion among the nanoparticles and superior constancy. In agreement with this study, the Au/cellulose nanocomposite biofabricated by green alga *Chlorella vulgaris* is negative (−13.6 mV) [78]. The difference in zeta potential values of these results and other research may be due to the zeta potential of cellulose nanocrystal (CNC) aqueous dispersions, as was the function of solution conditions, including changing pH and different electrolyte identities and concentrations [79].

### 3.6. Differential Scanning Calorimetry (DSC)

**Figure 8:**
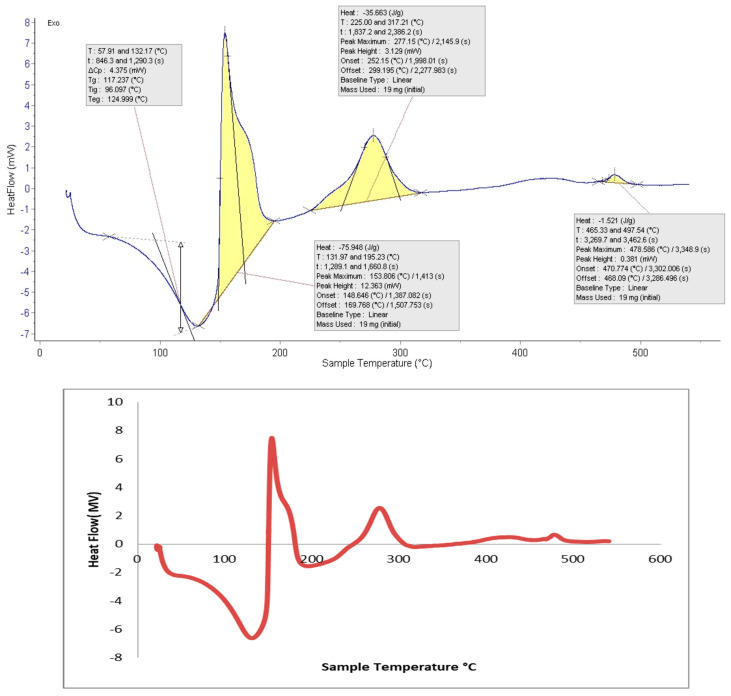
DSC of Ulva/Ag/cellulose nanocomposite blend with fluoride.

Figure 8 displays the DSC thermogram of Ulva/Ag/cellulose nanocomposites blended with fluoride, the temperature ranged from 20 °C to 600 °C. The glass transition temperature (Tg) 117.237 °C, was attained. The exothermic exhibited in the range of 148.648 and 169.788 °C may be due to the result of water evaporation. Composites exhibited initial decomposition around 100 °C, which could be due to the result of water evaporation [80]. According to the DSC curve, drug dehydration occurred between 50 and 121.8 °C [81]. In the second exothermic peak, ranging from 225 and 317.12 °C, thermal decomposition of cellulose nanocomposites was exhibited. The results are in agreement with Peter and Chrebet’s [82] reported cellulose decomposition beginning at temperatures of 250–260 °C. The results demonstrated the presence of exothermic peak during the heating scan of differential scanning calorimeter (DSC) analysis, called the cold crystallization peaks [83].

### 3.7. Antimicrobial Activity

The agar-well diffusion technique was used to investigate the antibacterial activities of hybrids made of AgNPs, Ulva/nanocellulose, and Ulva/Ag/cellulose nanocomposite at different concentrations of nanocellulose (0.1, 0.2, 0.4, and 0.8 g) against both *S. mutans* ATCC 25175 and *L. acidophilus* CH-2.

**Figure 9:**
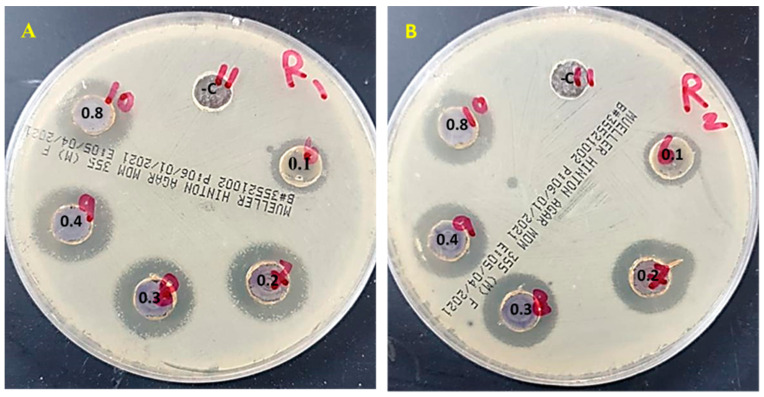
Antimicrobial activity of control, Ulva/nanocellulose, and Ulva/Ag/cellulose nanocomposite at different concentrations of cellulose (0.1, 0.2, 0.4, and 0.8 g) against both (**A**) *S. mutans* ATCC 25175 (R1) and (**B**) L. acidophilus CH-2 (R2) Clear zones (11), control Ulva extract, (6) Ulva/nanocellulose, clear zone of different cellulose concentrations (0.1, 0.2, 0.4, and 0.8 g) of Ulva/Ag/cellulose nanocomposite (7, 8, 9, and 10), respectively.

Results obtained from the clear zone (mm) around the two bacterial types revealed that all examined nanoparticles have antibacterial activity on selected indicator organisms, however, Ulva/nanocellulose showed low antibacterial activity. Both AgNPs and Ulva/Ag/cellulose nanocomposite showed greater inhibitory zone diameter than that obtained by Ulva/nanocellulose. Furthermore, no significant differences in inhibition zone diameter were observed when different cellulose concentrations were applied to process Ulva/Ag/cellulose nanocomposites (Figure 9 and Table 3). The interaction and toxicity of nanoparticles are more significant with the bacterial surface due to their small size and high surface area. [23]. Results in Figure 8 and Table 3 demonstrate Ulva/nanocellulose possessed low antibacterial activities against both *S. mutans* ATCC 25175 and *L. acidophilus* CH-. Nanocellulose, by nature, does not have any antimicrobial properties and needs surface modification to make it an antimicrobial material [84]. Khulbe and Matsuura [85] recorded metal/metal oxide nanoparticles (such as Cu, Au, CuO, ZnO, Ag, TiO_2_, and MgO), silanes, chlorine, and chitosan as coupling agents that provide nanocellulose with antimicrobial properties. In a previous study, nanocellulose was modified by combining it with chitosan to inhibit the growth of *Escherichia coli* by 99% [86]. Oxide/cellulose nanocomposite presented afflicted antibacterial activity against *Staphylococcus aureus* and *E. coli* [27]. The AgNPs cellulose composite showed excellent antibacterial activity to prohibit bacterial infection and biofilm formation efficacy against most types of bacteria [87]. These nanoparticles have different mechanisms of action, including damaging the bacterial cell membrane by transference of Ag^0^ to Ag^+^, as well as the obstruction of the intercellular metabolic pathways following Ag^+^ penetration into the cell [88]. Antibacterial experimental results showed that the cellulose–silver hybrids showed excellent antimicrobial activities against *E. coli* (Gram-negative) and *S. aureus* (Gram-positive) [70]. Since they exhibit antibacterial properties, CNF/ZnO films can be utilized in biological applications [89]. The antibacterial activity of silver against *K. pneumonia* strains (Gram-negative bacteria) and *S. aureus* (Gram-positive bacteria) has been reported [90]. Silver ions change the function of the bacterial wall after binding with it [91]. El-Abd et al. [92] reported that coating biosynthesis AgNPs with acetic acid reduced the microflora in Ross broiler chicks such as *Pseudomonas orizihabitain*. Hamouda et al. [93] estimated that biosynthesis AgNPs using two red algae capping SDS possessed antibacterial activities against *Micrococcus leutus*, *Kocuria varians*, and *Escherichia coli* ATCC 8739. Silver nanoparticles biofabricated by *Oscillatoria limnetica* demonstrated the highest antibacterial activity against multidrug-resistant bacteria (MDR) [77].

### 3.8. Antimicrobial Activity of Nanocellulose Blended with Fluoride

The results presented in Table 4 show that fluoride had no inhibition zone present with two bacterial strains, while nanocellulose blended with fluoride was more effective than nanocellulose and Ulva/Ag/cellulose nanocomposites/fluoride in the case of *S. mutans* ATCC 25175. Ulva/Ag/cellulose nanocomposites/fluoride possessed more activity than nanocellulose/fluoride against *L. acidophilus* CH-2. Fluoridated polyethylene glycol-coated silver nanoparticles (PEG-AgNPs) can be used for antibacterial activity against *S. mutans* [94]. Silver nanoparticles blended with fluoride had antibacterial activity against *Streptococcus mutans* [95]. In comparison with sodium fluoride, silver nanofluoride was more effective in inhibiting pH lowering and adherence of *S. mutans* to the enamel surface [96]. Widakdo et al. [97] reported graphene oxide (GO) had antibacterial qualities against *Escherichia coli*, and the antibacterial level increased to 99.9% in almost all membranes with different pH values, due to the synergistic effect between AgNPs, GO, GO-AgpHx that showed a lower molecular transmission resistance and a higher antibacterial effect. Linear low-density polyethylene (LLDPE) combined with CaO and metal ion forms (LLDPE/CaO Ag, LLDPE/CaO Zn, and LLDPE/CaO Cu) were excellent and functioned as antibacterial agents against *E. coli* with an antibacterial rate of 99.9% [98].

### 3.9. Minimum Inhibitory Concentration (MIC)

**Figure 10:**
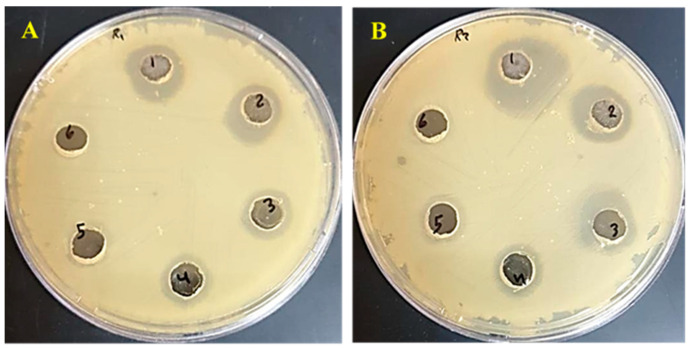
Effect of different concentrations (mg/mL) of Ulva /Ag/cellulose nanocomposites on *S. mutans* (**A**) and *L. acidophilus* (**B**).

The MIC values of Ulva/Ag/cellulose nanocomposites were tested against *S. mutans* ATCC 25175 and *L. acidophilus* CH-2 at concentrations ranging from 0.0018 to 0.000056 g/mL. The inhibition zone diameter values (mm) were dose dependent and gradually decreased by decreasing the concentration of Ag/Ulva/cellulose nanocomposites (Table 5 and Figure 10). The results show that the MIC of both bacteria was 0.000112 g/mL (0.112 mg/L). Hamouda et al. [92] reported that the MIC of AgNPs synthesis by marine green alga *Ulva fasciata* is 0.5 mg/mL for *S. aureus*, 1.0 mg/mL for *Salmonella enterica* sub sp., 0.5 mg/mL for *Aeromonas hydrophila*, 2 mg/mL for E. coli O157, and 1.0 mg/mL for *Bacillus cereus.* There are many studies that have proved the antibacterial activities of AgNPs derived from biomaterials and Ag cellulose nanocomposites against different bacteria, as tabulated in Table 6.

## 4. Conclusions

Nanocomposites have led to hurriedly increasing applications in various fields. This study investigated the effect of nanocellulose and *Ulva*/Ag/cellulose nanocomposites derived from marine alga *Ulva lactuca* against S. mutans *ATCC 25175* and *L. acidophilus CH-2.* Ulva/nanocellulose and Ulva/Ag/cellulose nanocomposites were characterized by TEM, SEM, zeta potential, EDX, XRD, and FT-IR. SEM confirmed that nanocellulose comprises filaments, and *Ulva*/Ag cellulose nanocomposites have fibrous surfaces. TEM images showed that the diameter of *Ulva*/nanocellulose ranged from 17 to 28 nm, while the diameter size of *Ulva*/Ag cellulose nanocomposites was 18.59 to 38.11. XRD confirmed that both *Ulva*/nanocellulose and *Ulva*/Ag cellulose nanocomposites were crystalline. Zeta potential indicated that *Ulva*/Ag cellulose nanocomposites were more negative in charge than *Ulva*/nanocellulose. *Ulva*/nanocellulose and *Ulva*/Ag cellulose nanocomposites have antibacterial activity against S. mutans and L. acidophilus. Data analysis revealed that Ulva/Ag/cellulose nanocomposites are more effective than *Ulva*/nanocellulose, and Ulva/Ag/cellulose nanocomposites blended with fluoride had more antibacterial activity than those not blended with fluoride. The antibacterial activities showed nonsignificant differences based on using different cellulose concentrations. Based on the data obtained, it is possible to deduce that Ulva/Ag/cellulose nanocomposites have the potential to be an economical and safe nanocomposite product from a natural source with antibacterial components that might be employed against bacteria that cause dental decay, blended to toothpaste, and add to tooth filler.

## Figures and Tables

**Table 1:** Tabulated absorption peaks assigned to the active groups of cellulose (A), nanocellulose (B), and Ulva/Ag/cellulose nanocomposites (C).

Wavenumber cm^−1^	A	B	C	Active Groups	References
3508	D	ND	ND	O-H stretching	[39,40]
3413	ND	D	+6	symmetric NH_2_	[41]
2924	D	ND	+4	C-H stretching	[42,43]
2851	D	+6	ND	CH_2_ symmetric	[44]
1718	ND	D	ND	C=O	[45]
109	ND	D	ND	C=O stretching	[46]
1656	D	ND	ND	Amide I	[47]
1638	D	−3	+1	amide I	[43,48]
1545	ND	D	+3	Peptide amide II	[49]
1529	ND	D	ND	amide II	[50]
1427	D	−24	ND	CH_3_	[51]
1382	ND	D	+2	CH bending vibrations	[52]
1233	ND	D	+1	PO2-asymmetric	[53]
1197	ND	D	ND	carbohydrates	[54]
1159	ND	D	+1	(C-C/C-N stretching)	[55,56]
1114	ND	D	ND	O-H association band	[57]
1037	D	+36	+36	C=O stretch	[51]
848	D	ND	ND	C-H	[58]
793	D	ND	ND	C-C bond	[59]
669	ND	D	D	C-H bending	[60]
607	D	−5	−5	C≡C-H	[61]
528	ND	D	D	C-S stretching	[61,62]

D: Detected, ND: Not Detected, (−): shifted wavenumber by minus, (+) shifted wavenumber by addition.

**Table 2:** Simple peak indexing of Ulva/nanocellulose and Ulva/Ag/cellulose nanocomposites.) derived from *U. lactuca*.

**S. No.**	**Peak Position 2θ**	**d-Spacing (Å)**	**hkl**	**Crystal Size L (nm)**	**Intensity %**
1	10.8	8.14658	200	17.2683	27.72
2	11.5	7.66832	(210)	8342	41.83
3	15.1	5.85977	(220)	48	17.57
4	20.6	4.30754	(400)	26	62.3
5	21.9	4.05492	(330)	8454	49.84
6	22.9	3.87688	(331)	8464	41.9
7	25.4	3.49637	(422)	3.8	23.61
8	26.5	3.3611	(431)	10.17	29.41
9	27.9	3.19667	(432)	38.79	36.88
10	29	3.07829	(440)	28.07	41.9
11	31.6	2.83081	(610)	27.83	100
12	33.5	2.67504	(443)	96.32	13
13	34.6	2.58812	(622)	96.62	12.72
14	37.6	2.39224	(551)	97.43	13.66
15	40.4	2.22844	(559)	74.24	2.22844
16	43.1	2.09515	(733)	117	2.09515
17	45.5	1.99357	(831)	9001	1.99357
18	48.1	1.89097	(911)	101	1.89097
19	50.2	1.81672	(762)	35.6	1.81672
**S. No.**	**Peak Position 2θ**	**d-Spacing (Å)**	**hkl**	**Crystal Size L (nm)**	**Intensity %**
1	11.5	7.68364	(000)	42.39	5.58
2	20.6	4.29569	(100)	35.72	10.51
3	23.2	3.82409	(110)	13.26	3.15
4	27.7	3.21829	(111)	28.96	45.55
5	28.9	3.08935	(111)	39.59	6.15
6	29.5	3.02172	(111)	72.69	0.95
7	30.9	2.88848	(111)	33.67	10.64
8	31.6	2.8291	(111)	73.84	32.05
9	32.1	2.78552	(111)	31.35	100
10	36.2	2.47933	(200)	49.32	2.42
11	37.2	2.41513	(210)	44.51	2.83
12	43.3	2.08628	(211)	25.22	1.3
13	45.4	1.99737	(211)	30.48	20.18
14	47.5	1.91105	(211)	19.21	1
15	49.0	1.85672	(220)	77.26	0.87
16	54.6	1.67844	(310)	27.93	14.53
17	55.8	1.64638	(310)	47.74	1.01
18	56.4	1.62887	(310)	29.93	4.54
19	57.3	1.60754	(310)	32.05	13.84
20	58.2	1.58487	(311)	60.36	0.6
21	66.0	1.41362	(320)	55.92	1.66
22	67.3	1.38953	(321)	41.65	5.48

**Table 3:** Inhibition zone diameter (mm) of *S. mutans* ATCC 25175 and *L. acidophilus* CH-2 at different concentrations of cellulose percentage to form Ulva/Ag/cellulose nanocomposites, Ulva/AgNPs, and nanocellulose derived from *U. lactuca*.

Strains	B	A	(C) with Cellulose Conc., %				Control
			1.6	0.8	0.4	0.2	
*S. mutans*	18 ± 0.2 c	18 ± 0.1 c	18 ± 0.2 c	19 ± 0.1 c	16 ± 0.00 b	13 ± 0.2 a	0
*L. acidophilus*	17 ± 0.13 c	18 ± 0.1 c	18 ± 0.05 c	18 ± 0.05 c	15 ± 0.1 b	13 ± 0.1 a	0

Nanocellulose 4 mg/mL (A); Ulva/AgNPs, 1.7 mg/mL (B); Ulva/Ag/cellulose nanocomposites (C). Different letters denote significance value at *p* < 0.05. Control: Ulva water extract.

**Table 4:** Inhibition zone diameter (mm) of *S. mutans ATCC 25175* and *L. acidophilus CH-2* of Ulva/cellulose and Ulva/Ag/cellulose nanocomposites that were blended with fluoride. (Different letters denote significance value *p* < 0.05).

Bacterial Strain	Nanocellulose	Ulva/AgNPs	Fluoride	Nanocellulose/Fluoride	Ulva/Ag/Cellulose Nanocomposites/Fluoride
*S. mutans*	13 ± 0.2 a	16 ± 0.00 b	0	23 ± 2 c	22 ± 1 c
*L. acidophilus*	13 ± 0.2 a	15 ± 0.1 b	0	23 ± 1 c	24 ± 2 c

**Table 5:** Inhibition zone (mm) of *S. mutans ATCC 25175* and *L. acidophilus* CH-2 at different concentrations of Ag/Ulva cellulose nanocomposites (g/mL) after 24 h.

Nanocomposites (g/mL)	0.0018	0.0009	0.00045	0.000225	0.000112	0.000056
*S. mutans*	19 ± 0.1	18 ± 0.3	15 ± 0.1	14 ± 0.00	11 ± 0.00	0±
*L. acidophilus*	19 ± 0.00	18 ± 0.1	15 ± 0.1	13 ± 0.00	12 ± 0.00	0±

**Table 6:** Comparison study with other researches on antibacterial activity.

	Sources	Antibacterial Against	Reference
AgNPs	*Sargassum wightii*	*Micrococcus luteus*, *Serratia marcescens*	[99]
	*Caulerpa serrulata*	*E. coli*, *Salmonella typhi*	[100]
	*Caulerpa racemosa*	*Staphylococcus aureus*, *Proteus mirabilis*	[101]
	*Chlorella ellipsoidea*.	*S. aureus*, *P. aeruginosa*, *K. pneumonia*, *E. coli*	[102]
	*Ecklonia cava*	*E. coli*	[103]
AgNPS/Cellulose	Orange peel waste	*E. coli*	[104]
	Cotton pulp cellulose	*E. coli*, *P. aeruginosa*, *S. aureus*	[105]
	Bacterial cellulose	*B. subtilis*, *S. aureus*, *E. coli*	[106]
	Bacterial cellulose	*Escherichia coli* *Staphylococcus aureus*	[107]
	Ag/Ulva cellulose nanocomposites	*S. mutans* ATCC 25175 and *L. acidophilus* CH-2	This study

## Data Availability

The datasets spent and/or analyzed during this study are available from the corresponding author upon reasonable request.
